# Special Analytical and Health Commission on Tinned and Preserved Food

**Published:** 1906-07-07

**Authors:** 


### SPECIAL ANALYTICAL AND HEALTH COMMISSION ON TINNED AND PRESERVED FOOD.

### II. PRESERVED MEAT FOODS PREPARED IN ENGLAND.

### More Sausages.

Since concluding our article upon this subject last week we have received several other samples of sausages. Concerning the average chemical composition and dietetic value of sausages, we must refer our readers to last week's article, as we simply purpose here describing the properties of the actual samples that have been submitted to us. We have received five samples of sausages from Messrs. Palethorpes', Limited, of Dudley Port, Staffs. The Cambridge (pork) sausages of this firm are guaranteed pure English, and after careful examination we have come to the conclusion that they are of excellent quality. What appears to be a speciality of this maker is tomato sausages. These are ordinary sausages in the substance of which tomatoes, or rather tomato pulp, is intermixed. To those who like the taste of tomatoes, these sausages will form a pleasant addition to their diet and an agreeable change from the ordinary sausage. We regard the incorporation in the sausage of a fresh vegetable pulp as a distinct advance in sausage-making technique, and think this article is distinctly to be recommended. The same makers have also sent us their smoked Cambridge sausages. We have nothing further to say of these, except that we regard them of first-class quality and equal to any smoked sausage we have examined. The fourth kind of sausages from this firm is a ham, chicken, and tongue sausage. This type of sausage, as we have remarked before, must be regarded more as a concentrated meat preparation. It contains less moisture than the ordinary sausage, and less fat is cooked, and will keep much longer and under more unfavourable conditions. Messrs. Palethorpe have also forwarded one pound of polonies, and a one-pound pork pie. We regard both these articles as being well up to the usual standard, and perfectly wholesome. In our last issue we referred to the sausages forwarded to us for examination by Messrs. Harris. These were from William Harris, digestive sausage manufacturer, West Smithfield. They can be described as useful and wholesome articles of diet.

### Soups, Beef Teas, and Meat Extracts.

St. Ivel high-class chicken broth soup, prepared by Aplin and Barrett, etc., Ltd., Yeovil, Somerset, England. This soup is prepared from chicken broth, to which bits of chicken meat and pearl barley have been added. It will thus be seen that it is prepared on the principle of Scotch broth, except that the basis is chicken. It is needless to say that the addition of these substances to the soup greatly increases its nutritive value, and the preparation before us is essentially not only a stimulant, but a food. The bottle of soup sent to us has kept well under ordinary domestic conditions, and when one-third of its volume of water is added to it, and it is kept at the boil for five minutes, it makes a pleasant soup.

Messrs. Benoist have sent us a bottle of their *consommé.* This preparation remains semi-solid and transparent at the present summer temperature. When heated carefully with the addition of water it makes a delicious *consommé,* which may be still further improved by the use of a small quantity of a suitable wine. This *consommé* is well up to the high standard of the other preparations by this maker. It is sent out in wide-necked bottles.

The Oxvil Company have supplied a bottle of Oxvil. This substance is used in the strength of one teaspoonful to a "cup" of boiling water. It is guaranteed as made from fresh ox beef only. It is one of the preparations made on the principle of extracts of meat to which the albumen and fibrin is either subsequently added or retained in the process of manufacture. Hence it contains not only stimulant meat bases, but also nutrient material.

The Australian Meat Company (Ramornie) have submitted a 2 oz. pot of Liebig's Extract of Meat. This extract of meat is prepared according to the original technique of Professor Liebig, and must be regarded, as Liebig originally said of his extract, as a stimulant—like tea or coffee, and not as a food. Ample directions are given on the pot for the making of soups and beef teas by means of this extract with or without the aid of other nutritive materials. The extract possesses an excellent flavour, and contains an adequate amount of meat bases which show the genuineness of its origin and its manufacture. We have received two other brands of Liebig's extract—namely, one from Messrs. Lazenby and one from Messrs. F. L. Borthwick and Co. What we have said of the former brand of Liebig's extract applies equally to these two latter ones. They are distinctly of value in invalid dietetics, and also for imparting an appetising aroma and flavour to soups and broths which would otherwise, however nutritious they may be, fall flat on the palate and probably fail to excite reflexly those physiological phenomena upon which their digestion and absorption, and hence their value as foods, depend.

Messrs. Mason and Co., Limited, Chelsea Works, Walham Green, London, S.W., have sent us various samples from their works. We have examined carefully their essence of beef. This preparation, which is in the form of a jelly, consists solely of the juice of the finest English beef. We think this beef jelly, which should be taken by the patient in jelly form, is equal to any beef essence on the market, and is to be strongly recommended. The same firm have submitted to us three other broths or meat teas specially prepared for invalids—namely, veal broth, mutton broth, and savoury beef tea. These preparations are all excellent, and simply require heating to be ready for use. We are very pleased to know that there is on the market a reliable veal tea. Veal tea as opposed to beef tea is exceedingly useful in certain cases of intestinal irritation, especially in the chronic diarrhœa of children, and we are sure that now their attention has been directed to it, physicians will avail themselves of this preparation.

Messrs. Liebig's Extract of Meat Company, 4 Lloyds Avenue, E.C., have submitted to us a tin of their prime Fray Bentos clear ox tail soup. We think the name Fray Bentos given by this company to their preparations is an exceedingly apt one. It will be remembered by all cognisant of the history of meat extracts that, subsequent to the classical experiments made in the middle of the last century by Pettenkofer and Justus von Liebig, the manufacture of meat extracts upon a commercial scale was started at Fray Bentos, in Uruguay. The present company are the descendants of the commercial originators of the preparation of meat extract by Liebig's process, and although they have now no exclusive right to either the name or the process they certainly, to those who know anything of the subject, give unmistakable evidence of their descent by using the name Fray Bentos. When their clear ox tail soup is mixed with an equal quantity of water and seasoned to taste, a thoroughly pleasant and refreshing beverage results. We have received from Messrs. Palethorpe a tin of their English ox tail soup. In order to be prepared for consumption this soup must be heated with one-third its quantity of water. We have tried it, and found it in every way good. The inside of the tin we examined was perfectly bright, and there was no evidence of metallic contamination.

